# An online tool for evaluating diagnostic and prognostic gene expression biomarkers in bladder cancer

**DOI:** 10.1186/s12894-015-0056-z

**Published:** 2015-07-01

**Authors:** Garrett M. Dancik

**Affiliations:** Mathematics and Computer Science Department, Eastern Connecticut State University, Science Building, Rm. 257, Willimantic, CT 06226 USA

**Keywords:** Urothelial carcinoma, Gene expression profiling, Biomarkers, Bladder cancer

## Abstract

**Background:**

In the past ~15 years, the identification of diagnostic and prognostic biomarkers from gene expression data has increased our understanding of cancer biology and has led to advances in the personalized treatment of many cancers. A *diagnostic* biomarker is indicative of tumor status such as tumor stage, while a *prognostic* biomarker is indicative of disease outcome. Despite these advances, however, there are no clinically approved biomarkers for the treatment of bladder cancer, which is the fourth most common cancer in males in the United States and one of the most expensive cancers to treat. Although gene expression profiles of bladder cancer patients are publicly available, biomarker identification requires bioinformatics expertise that is not available to many research laboratories.

**Description:**

We collected gene expression data from 13 publicly available patient cohorts (*N* = 1454) and developed *BC-BET,* an online *B*ladder *C*ancer *B*iomarker *E*valuation *T*ool for evaluating candidate diagnostic and prognostic gene expression biomarkers in bladder cancer. A user simply selects a gene, and *BC-BET* evaluates the utility of that gene’s expression as a diagnostic and prognostic biomarker. Specifically, *BC-BET* calculates how strongly a gene’s expression is associated with tumor presence (distinguishing tumor from normal samples), tumor grade (distinguishing low- from high-grade tumors), tumor stage (distinguishing non-muscle invasive from muscle invasive samples), and patient outcome (e.g., disease-specific survival) across all patients in each cohort. Patients with low-grade, non-muscle invasive tumors and patients with high-grade, muscle invasive tumors are also analyzed separately in order to evaluate whether the biomarker of interest has prognostic value independent of grade and stage.

**Conclusion:**

Although bladder cancer gene expression datasets are publicly available, their analysis is computationally intensive and requires bioinformatics expertise. *BC-BET* is an easy-to-use tool for rapidly evaluating bladder cancer gene expression biomarkers across multiple patient cohorts.

**Electronic supplementary material:**

The online version of this article (doi:10.1186/s12894-015-0056-z) contains supplementary material, which is available to authorized users.

## Background

Cancer is a genetic disease [1]. A cancer cell inherits or acquires mutations that enable it to grow efficiently, replicate indefinitely, support angiogenesis, avoid apoptosis, and in some cases metastasize [2]. In the past ~15 years, gene expression profiling of human cancers has revolutionized our understanding of cancer as a genetic disease and has expedited the identification of driver mutations and biomarkers for personalized treatment. A *diagnostic* biomarker is a molecule that is indicative of cancer status, such as the existence of a tumor, or its stage, grade, or clinical subtype; a *prognostic* biomarker is indicative of disease outcome.

Examples of prognostic biomarkers in routine clinical use include the Onco*type*Dx and MammaPrint gene panels, which both predict the likelihood of disease recurrence in breast cancer and provide patients and clinicians with relevant information regarding the potential benefit of chemotherapy [3, 4].

In the United States, bladder cancer is the fourth most common cancer in males, the eighth most common cancer in females [5], and one of the most expensive cancers to treat [6]. At diagnosis, approximately 20–30 % of bladder cancer patients harbor muscle invasive tumors [7] and these patients have a five-year survival rate of approximately 43 % [8]. However, despite the importance of this disease there are no prognostic biomarkers or targeted therapies in clinical use.

Data from high-throughput gene expression studies (that simultaneously measure the expression of 1000s of genes) are typically deposited into public databases such as the Gene Expression Omnibus (GEO; Barrett and Edgar, 2006) and ArrayExpress [9]. These databases function primarily as data repositories where data is downloaded and analyzed using in-house bioinformatics tools. This analysis is often time-consuming and requires computational resources and bioinformatics expertise often not available to biologists or clinician-researchers. Furthermore, there are currently no bladder cancer gene expression databases that allow for an automated and comprehensive evaluation of both diagnostic and prognostic biomarkers in patients across multiple cohorts. In particular, the GEO2R tool allows for the identification of diagnostic biomarkers in any GEO cohort, but cannot identify prognostic biomarkers and cannot analyze multiple patient cohorts in a single analysis. Other databases include the KM plotter [10], which generates Kaplan-Meier survival curves for lung, breast, and ovarian cancer patients only; and PrognoScan [11] and SurvExpress [12], which generates Kaplan-Meier curves for only a small subset of the available bladder cancer patients. None of the above mentioned tools can identify prognostic biomarkers independent of stage or grade.

Here, we describe an online *Bladder Cancer Biomarker Evaluation Tool* (*BC-BET*) for rapid evaluation of diagnostic and prognostic biomarkers in bladder cancer, using data from 13 patient cohorts (*N* = 1454). The next section describes the patient cohorts and analyses that are implemented in *BC-BET*. We then demonstrate the utility of the tool by analyzing *FGFR3*, a gene known to be associated with tumor grade and stage in bladder cancer.

## Construction and content

### Patient cohorts and gene expression datasets

We performed a systematic search of the Gene Expression Omnibus (GEO) [13] and Array Express [9] and identified all entries with the keyword “bladder cancer” whose data was obtained by “transcription profiling by array”. Cohorts were excluded if *all* patients received neoadjuvant or adjuvant chemotherapy. Of the patient cohorts identified, four cohorts (GSE88, GSE89, GSE7476, GSE27448) were then excluded due to an insufficient sample size for both diagnostic (<10 per group) and prognostic (<10 events) biomarker evaluation (see below). Eleven patient cohorts from GEO and ArrayExpress met our inclusion criteria. Two additional cohorts were identified following a literature search and downloaded from supplemental material to publication [14, 15]. In these cohorts, individual patients were excluded from the survival analyses if they were treated by neoadjuvant chemotherapy and excluded completely if their tumors did not have urothelial histology. Individual patients treated with intravesical therapy or adjuvant chemotherapy can be optionally excluded from the prognostic biomarker evaluation analysis (see *Prognostic biomarker evaluation and survival analysis*). In all, *BC-BET* contains 13 patient cohorts (*N* = 1454) profiled on 10 distinct microarray platforms. Cohorts are named according to the institution or individual responsible for collecting and uploading the dataset, and are summarized in Table 1. All patient samples were collected with approval from an appropriate Institutional Review Board, as documented in the original publications (see Table 1).

**Table 1:** The 13 patient cohorts (*N* = 1454) included in *BC-BET.* The numbers in the table correspond to the number of patients with each clinical characteristic or available endpoint that are included in the database and analyzed. A ‘-’ denotes insufficient sample size for analysis

		# of samples						
Cohort (availability)*	Platform	Normal, Tumor	LG, HG	NMI, MI	DSS	OS	RFS	Total (N)
AUH-1 [27] (GSE3167)	Affymetrix Human Genome U133A	9,41	8, 32	28, 13	–	–	–	50
AUH-2 [28] (GSE5479)	MDL Human 3 k	–	98, 271	351, 51	–	–	–	404
Blaveri [14] (S)	UCSF Human Array 2.0	–	10, 68	27, 53	–	74	–	74
CNUH [29] (GSE13507)	Illumina human-6 v2.0	10, 165	105, 60	104, 61	165	165	–	175
DFCI [30] (GSE31684)	Affymetrix Human Genome U133 Plus 2.0	–	6, 84	15, 78	–	–	90	93
Lindgren [31] (GSE19915)	Swegene	12,144	72, 72	97, 45	–	142	–	156
Lindgren-2 [32] (GSE32548)	Illumina HumanHT-12 V3.0	–	56, 75	92, 38	–	89	–	131
MDA-1 [33] (GSE48276)	Illumina HumanHT-12 WG-DASL V4.0 R2	–	–	–	–	22^†^	–	22
MDA-2 [33] (GSE48075)	Illumina HumanHT-12 V3.0	–	–	67, 73	–	73^†^	–	140
MSKCC [15] (S)	Affymetrix Human Genome U133A	38,91	18, 73	25, 66	87	–	–	129
UVA [34] (GSE37317)	Affymetrix Human Genome U133A	–	–	8, 10	–	–	–	18
Stransky-1 [35] (E-TABM-147)	Affymetrix Human Genome U95A	5,26	11, 15	9, 17	–	–	–	31
Stransky-2 [35] (E-TABM-147)	Affymetrix Human Genome U95Av2	–	13, 16	16, 15	–	–	–	31
Total		74, 467	397, 769	839, 523	252	565	90	1454

*Gene expression data for all cohorts are publicly available from the Gene Expression Omnibus (GEO) [13] with the given Accession # (GSE ID), from Array Express [9] (Accession # E-TABM-147) or as Supplementary material to publication (S). ^†^patients have MI, HG tumors (MDA-1) or MI tumors with unspecified grade (MDA-2). Abbreviations: LG, low grade; HG, high grade; NMI, non-muscle invasive; MI, muscle-invasive; DSS, disease-specific survival; OS, overall survival; RFS, recurrence-free survival

For each cohort, the processed gene expression data was downloaded. Three cohorts (Blaveri, AUH-2, and MDA-1) had missing values. Microarray probes with missing values in >20 % of samples were removed and expression values imputed using the *impute* package (*impute.knn* function) in *R* with default parameters. Probes are matched to genes based on current microarray (e.g., Affymetrix) annotation. When multiple probes exist for a gene, the probe with the highest mean expression is used [16]. In cohorts with replicate samples (AUH-2, MSKCC, Stransky-1, and Stransky-2), replicate samples were averaged to produce a single gene expression profile for each patient. In the MDA-1 cohort, because only two individuals had NMI tumors, the two individuals with NMI tumors are excluded from the database. When the same patient was profiled in multiple cohorts, and both cohorts are analyzed (i.e., the gene of interest is profiled in both cohorts), then duplicate patients are removed from one of the cohorts in order to prevent biasing of the results. For details, and for more information about the patient cohorts, sample processing, and patient exclusion, see Additional file 1: Table S1.

### Diagnostic biomarker evaluation

For a specified gene, *BC-BET* evaluates whether the candidate gene is differentially expressed between the following groups which we denote as group *A* and group *B*, respectively: tumor and normal samples; high-grade (HG) and low-grade (LG) tumors; and muscle invasive (MI) and non-muscle invasive (NMI) tumors. LG and HG tumors correspond to G1-G2 and G3, respectively, or are classified according to the low- vs. high-grade classification system [17, 18]. NMI and MI tumors correspond to Ta-T1 or T2-T4 tumors, respectively [19]. Group differences are quantified by fold-change (FC) or by the area under the receiver operating characteristics curve (AUC). FC is calculated using the formula

$$ FC=\frac{mean\  expression\  of\  group\ A\  samples}{mean\  expression\  of\  group\ B\  samples} $$

The AUC quantifies the ability of gene expression to discriminate between two groups and is equivalent to the probability that a randomly selected sample from group *A* has higher expression than a randomly selected sample from group B. We note that AUC is a better measure of a biomarker’s predictive value, while FC may have a more intuitive biological interpretation. Statistical significance is assessed by either the two-sample *t*-test (which tests the hypothesis that FC = 1) or the non-parametric Wilcoxon-Rank sum test (which tests the hypothesis that AUC = 0.50).

### Prognostic biomarker evaluation and survival analysis

Endpoints include disease-specific survival (DSS), overall survival (OS), or recurrence-free survival (RFS). The events for these endpoints are death from disease, death from any cause, and disease recurrence for DSS, OS, and RFS, respectively. Because there is no single endpoint consistently available across all cohorts with survival information, a user can opt to use the *Best Available* endpoint, which is taken to be the first available endpoint in the following order: DSS, OS, and RFS. The endpoints available for each cohort are listed in Table 1. Although selection of the *Best Available* endpoint results in the selection of different endpoints for different cohorts, patients with MI tumors have significantly (*P* < 0.05) poorer outcomes than patients with NMI tumors using the best available endpoint in 5/6 cohorts that include both NMI and MI tumors (Additional file 1: Table S2), which is consistent with RFS and OS analyses in a long-term study of over one thousand patients [8]. Patients with HG tumors also have significantly (*P* < 0.05) poorer outcomes than patients with LG tumors in all cohorts with more than 10 NMI tumors (Additional file 1: Table S2). Therefore, stage and grade are consistently associated with outcome in these diverse cohorts when the *best available* endpoint is used.

With the exception of CNUH and DFCI, definitive treatment of all patients was either transurethral resection of the bladder or radical cystectomy. In CNUH, 56 patients with NMI tumors received intravesical Bacillus Calmette-Guerin therapy and 26 patients with MI tumors received cisplatin-based adjuvant chemotherapy (along with one patient with an NMI tumor). Because neither of these treatments are associated with outcome in this cohort (Additional file 2: Figure S1A-B), these patients are included by default in the survival analysis, but optionally can be excluded. In DFCI, there is a confounding between TNM staging and treatment with chemotherapy. In particular, 32 patients with MI tumors receive adjuvant chemotherapy while 1 patient with an NMI tumor receives adjuvant chemotherapy. Of the treated patients having MI tumors, 84 % have nodal involvement or distant metastases, and treated patients have a higher risk of recurrence than non-treated patients (Additional file 2: Figure S1C). The user can optionally remove these treated patients from the survival analysis if desired.

Survival analysis is carried out using the *coxph* function in *R* to obtain a hazard ratio (HR) and log-rank p-value based on the *cutpoint* selected by the user. If the *median* cutpoint is selected, patients are separated into two groups according to those with high expression (≥ the median) and low expression (< the median), and the HR corresponds to the hazard rate of the high expressers relative to the hazard rate of the low expressers. Alternatively, the *continuous* expression value of the gene may be used, in which case the HR is relative to a two-fold increase in gene expression.

## Utility and discussion

*BC-BET* was developed to allow for a systematic and comprehensive evaluation of candidate diagnostic and prognostic biomarkers across publicly available bladder cancer gene expression datasets. The user selects a gene, and *BC-BET* evaluates whether the gene of interest is a robust biomarker for tumor status, grade, and stage, based on its expression. For diagnostic biomarkers, the user selects the *class comparison measure* (FC or AUC) to quantify differences between tumor and normal samples, HG and LG samples, and MI and NMI samples.

To evaluate the utility of a gene as a prognostic biomarker, the user selects the desired endpoint (DSS, OS, RFS, or *Best Available*; see Construction and Content). Three analyses are then carried out: an evaluation of the prognostic value of the gene in cohorts containing patients with both NMI and MI tumors, in patients containing only LG, NMI tumors in each cohort, and in patients containing only HG, MI tumors. The latter analyses are critical for evaluating whether a biomarker has prognostic value independent of stage and grade, and are a unique feature of *BC-BET*. We note that there is one cohort (MDA-2) containing patients with MI tumors and unknown grade status, and the results from this cohort are displayed along with the results for patients with HG, MI tumors.

### Case study: evaluation of FGFR3

In order to demonstrate the functionality of *BC-BET*, we will evaluate the diagnostic and prognostic value of fibroblast growth factor receptor 3 (*FGFR3*). Genomic studies in bladder cancer suggest that MI and NMI tumors arise through divergent genomic pathways [20] and may arise from distinct progenitor cell types [21]. Specifically, activating *HRAS* and *TP53*/*RB1* mutations are more prevalent in MI tumors while *FGFR3* and *KDM6A* mutations are more prevalent in NMI and LG tumors [22–26]. Furthermore, FGFR3 mutation status is positively correlated with FGFR3 expression [25]. We therefore expect high *FGFR3* mRNA expression to be associated with NMI and LG tumors in *BC-BET*.

**Fig. 1:**
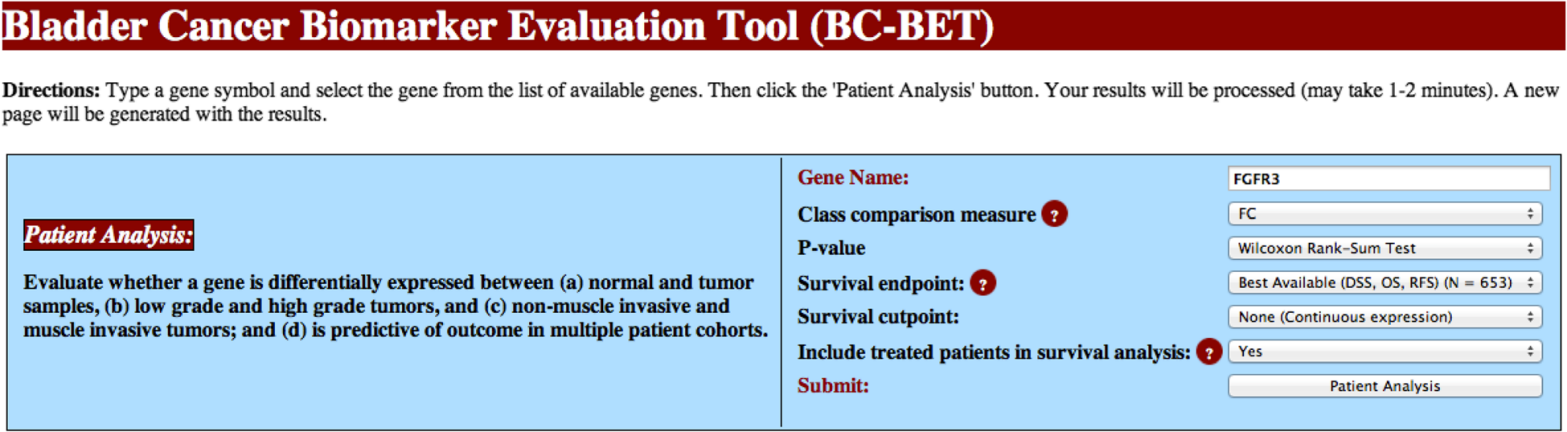
Screenshot of *BC-BET* database. The user selects the gene symbol (*FGFR3* is shown) from a dropdown list of available genes, and specifies additional parameters for the analysis. Here, class comparisons are quantified by fold change (FC), *p*-values will be calculated by the non-parametric Wilcoxon Rank-Sum test, and survival analysis will use the *Best Available* end point (see Construction and Content) and the continuous gene expression value, and treated patients will be included in the survival analysis in the CNUH and DFCI cohorts. The user clicks on *Patient Analysis* to evaluate the gene

A screenshot of the *BC-BET* homepage is provided in Fig. 1. Here the user selects the gene symbol of interest from a drop down menu of available genes, and sets the statistical parameters for the analysis. For class comparison, we will use FC to measure differential expression across tumor status, grade, and stage, and calculate *p*-values using the Wilcoxon Rank-Sum test. For survival analysis, we will look at continuous *FGFR3* expression using the *Best Available* endpoint. The user also has the option of including or excluding treated patients from the CNUH and DFCI cohorts. Clicking on the *Patient Analysis* button carries out the desired evaluation.

**Fig. 2:**
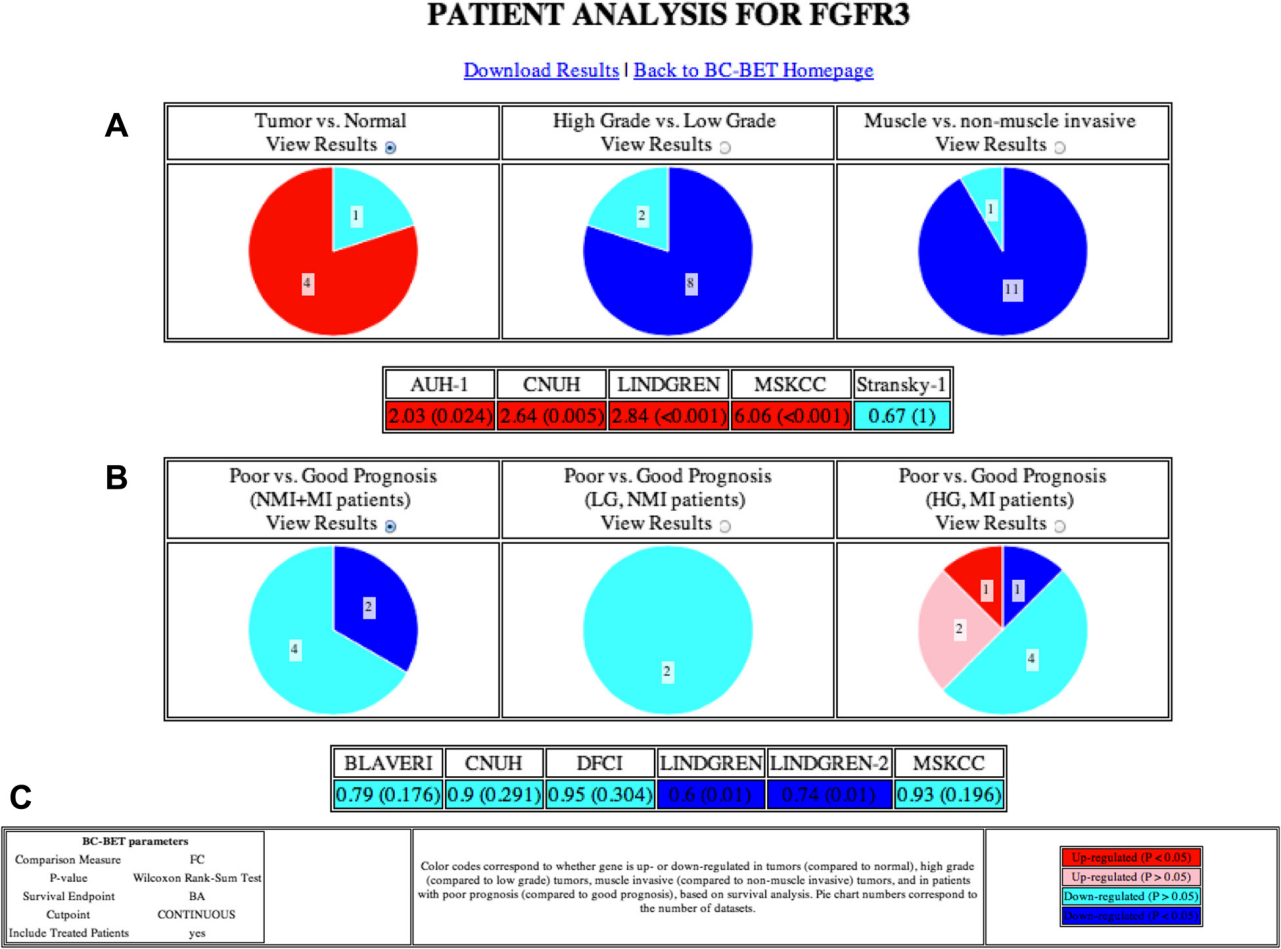
Screenshot of *BC-BET* analysis of *FGFR3*. The (**a**) diagnostic and (**b**) prognostic value of the gene is summarized graphically across the available cohorts. The results are color coded according to whether gene expression is (**a**) significantly (*P* < 0.05) up-regulated (*red*) or down-regulated (*blue*), or not significantly (*P* > 0.05) up-regulated (*pink*) or down-regulated (*light blue*), in normal, high grade, or non-muscle invasive samples (compared to tumor, low grade, and muscle invasive samples, respectively); and whether gene expression is (**b**) significantly (logrank *P* < 0.05) negatively (*red*) or positively (*blue*) associated with survival, or not significantly (*P* > 0.05) negatively (*pink*) or positively (*light blue*) associated with survival. Regions of each pie chart are labeled according to the number of cohorts with the corresponding result. (**c**) Summary of *BC-BET* parameters and legend

Figure 2 contains a screenshot summarizing the diagnostic (Fig. 2a) and prognostic (Fig. 2b) value of *FGFR3*. The diagnostic value of the gene is summarized and illustrated according to whether the gene is up-regulated (FC > 1) or down-regulated (FC < 1) in *group A* (i.e., tumor samples, HG tumors, or MI tumors) relative to *group B* (i.e., normal samples, LG tumors, or NMI tumors, respectively), and whether or not the result is statistically significant (*P* < 0.05). The prognostic value of the gene is summarized according to whether the gene is negatively (HR > 1) or positively (HR < 1) associated with survival, and whether or not the result is statistically significant (*P* < 0.05). A legend is included on the results page displayed by *BC-BET* (Fig. 2c).

Clicking on the appropriate *View Results* radio button provides detailed numeric results indicating the diagnostic value (FC and *p*-value) and prognostic value (HR and logrank *p*-value) for each cohort for the chosen analysis. By default, the “Tumor vs. Normal” and “Poor vs. Good Prognosis (NMI + MI patients)” detailed results are shown. The full set of results can be downloaded as an Excel spreadsheet by clicking the *Download Results* link at the top of the page (Additional file 3: Table S3).

From these results we see that *FGFR3* expression is significantly (*P* < 0.05) up-regulated in tumor samples (i.e., is higher in tumor samples than in normal samples) in 4/5 cohorts with this information, indicating its value as a diagnostic biomarker. *FGFR3* expression is also significantly (*P* < 0.05) down-regulated in HG tumors (i.e., is higher in LG tumors than HG tumors) in 8/10 cohorts, and significantly down-regulated in MI tumors in 11/12 cohorts with stage information. However, as a prognostic biomarker, *FGFR3* expression is only significantly (*P* < 0.05) associated with outcome in 2/6 cohorts, although in all cohorts it is positively associated with survival (HR < 1). In addition, *FGFR3* does not have any prognostic value independent of stage and grade, as it is not significantly associated with outcome in patients with LG, NMI tumors or consistently associated with outcome in patients with HG, MI tumors. The above results are consistent with previous studies showing that FGFR3 protein expression is associated with stage and grade in bladder cancer [25], and supports investigation into targeting *FGFR3* in patients with NMI tumors [20]. However, *FGFR3* expression has little prognostic value based on the available gene expression cohorts.

### Future developments

*BC-BET* will be updated periodically with additional gene expression datasets as they become available. In addition, new features including the ability to query the database with a list of genes or by probe, and the option to generate Kaplan-Meier curves are expected to be implemented in future versions of *BC-BET*.

## Conclusions

The identification of diagnostic and prognostic biomarkers based on gene expression data is a powerful approach for investigating cancer. Although many gene expression datasets are available, the comprehensive analysis of multiple patient cohorts requires bioinformatics expertise not always available to researchers. *BC-BET* is a *Bladder Cancer Biomarker Evaluation Tool* developed for the comprehensive and rapid evaluation of diagnostic and prognostic biomarkers in bladder cancer across multiple patient cohorts. However, *BC-BET* has several limitations. In particular, the available patient cohorts included here (Table 1 and Additional file 1: Table S1) are heterogeneous, profiled on multiple platforms, and lack a common endpoint for survival analyses. Although the identification of diagnostic or prognostic biomarkers in multiple patient cohorts is promising, any biomarker identified using *BC-BET* ultimately must be prospectively validated to determine its clinical utility. Nevertheless, the identification of robust biomarkers in bladder cancer will increase our understanding of this disease and may have important implications for treatment.

## Availability and requirements

*BC-BET* requires only a web browser and is available from Eastern Connecticut State University’s Bioinformatics Page: http://bioinformatics.easternct.edu/BCBET.

## Aditional files

Additional file 1: Table S1. Summary of patient cohorts, sample exclusion and processing for *BC-BET*. The *Complete cohort* columns correspond to all samples available at the given accession number or publication, while the *BC-BET* columns corresponds to the samples included in the database. **Table S2**. Association of stage (MI vs. NMI) and grade (HG vs. LG) with the *best available* endpoint. Statistically significant associations (*P* < 0.05) are highlighted in bold. Additional file 2: Figure S1. Association of treatment with outcome in CNUH and DFCI cohorts. Kaplan-Meier curves for CNUH cohort showing association of disease-specific survival (DSS) and overall survival (OS) with (A) intravesical Bacillus Calmette-Guerin (BCG) therapy in patients with NMI tumors and (B) cisplatin-based adjuvant chemotherapy in patients with MI tumors. C, Association of recurrence-free survival (RFS) with adjuvant chemotherapy in patients with MI tumors in DFCI chort. *P*-values are calculated by log-rank test. Abbreviations: HR, hazard ratio; N+, nodal involvement (pN1-pN3); M+, distant metastasis. Additional file 3: Table S3.
*BC-BET* results: evaluation of *FGFR3.* The spreadsheet consists of multiple sheets with a description of each sheet at the top. The TUMOR, GRADE, and STAGE sheets contain the results of the diagnostic biomarker evaluation comparing tumor vs. normal samples, HG vs. LG tumors, and MI vs. NMI tumors, respectively. The SURVIVAL, SURVIVAL.LG.NMI, SURVIVAL.HG.MI sheets contain the results of the prognostic biomarker evaluation in patients with both NMI and MI tumors, patients with LG, NMI tumors, and patients with HG, MI tumors, respectively.

## Abbreviations

LG: Low-grade
HG: High-grade
NMI: Non-muscle invasive bladder cancer
MI: Muscle invasive bladder cancer
FC: Fold-change
AUC: Area under the receiver operating characteristic curve
DSS: disease-specific survival
OS: Overall survival
RFS: Recurrence-free survival
HR: Hazard ratio
