# Supplementary material for: An online tool for evaluating diagnostic and prognostic gene expression biomarkers in bladder cancer
Source: BMC Urol. 2015 Jul 1;15:59. doi: 10.1186/s12894-015-0056-z (PMC4487975; doi:10.1186/s12894-015-0056-z)
Supplement: Additional file 1: Table S1. — Summary of patient cohorts, sample exclusion and processing for BC-BET. The Complete cohort columns correspond to all samples available at the given accession number or publication, while the BC-BET columns corresponds to the samples included in the database. Table S2. Association of stage (MI vs. NMI) and grade (HG vs. LG) with the best available endpoint. Statistically significant associations (P < 0.05) are highlighted in bold. [file 12894_2015_56_MOESM1_ESM.docx]

**Table S1.** Summary of patient cohorts, sample exclusion and processing for *BC-BET*. The ***Complete cohort*** columns correspond to all samples available at the given accession number or publication, while the ***BC-BET*** columns corresponds to the samples included in the database.

|  | ***Complete cohort*** | | ***BC-BET*** | |
| --- | --- | --- | --- | --- |
| Cohort (availability)* | N | Description | N | Sample Exclusion, Processing, and Additional Notes |
| AUH-1 [[1](#_ENREF_1)]  (GSE3167) | 60 | Forty-one tumor biopsies, 9 biopsies of normal bladder mucosa from patients without bladder cancer, and 5 paired samples consisting of a normal biopsy and a biopsy with CIS were profiled. | 50 | The paired biopsy samples are excluded. |
| AUH-2 [[2](#_ENREF_2)]  (GSE5479) | 808 | Tumors from 404 patients were obtained from bladder cancer patients from hospitals in Denmark, Sweden, England, Spain, and France and were profiled in duplicate. | 404 | Replicate profiles were averaged to produce a single gene expression profile for each patient. Eighteen samples are also profiled in AUH-1 and are removed if AUH-1 is analyzed. |
| Blaveri [[3](#_ENREF_3)] (S) | 80 | Eighty tumor samples were obtained from the tissue bank of the University of California at San Francisco Comprehensive Cancer Center. Six of these samples had squamous histology. All patients receive either transurethral resection of the bladder (TURBT) or cystectomy. Specifically, of those from transitional cell carcinomas, 25/27 patients with NMI tumors underwent TURBT while 44/47 patients with MI tumors receive cystectomies. | 74 | The 6 samples with squamous histology are excluded. For prognostic biomarker evaluation, only the NMI samples obtained from TURBT and MI samples obtained from cystectomy are analyzed. |
| CNUH [[4](#_ENREF_4)] (GSE13507) | 256 | Samples were collected at Chungbuk National University Hospital (CNUH), and consisted of 165 primary bladder cancer samples, 23 recurrent tumor samples, 58 normal samples surrounding tumors, and 10 normal bladder samples from patients with benign disease. Profiles from NMI tumors were obtained by transurethral resection of the bladder while those from MI tumors were obtained by cystectomy. Fifty-six patients with intermediate- or high-risk NMI tumors received intravesical Bacillus Calmette-Guerin therapy while 26 patients with MI tumors and 1 patient with an MI tumor received cisplatin-based adjuvant chemotherapy. | 175 | Samples from recurrent tumors and samples of bladder mucosae surrounding cancer are excluded. The user has the option of excluding patients treated with intravesical therapy or chemotherapy in the prognostic biomarker evaluation. |
| DFCI [[5](#_ENREF_5)]  (GSE31684) | 93 | Tumors were profiled from 93 patients undergoing radical cystectomy at Memorial Sloan Kettering Cancer Center between 1993 and 2004. Three patients received neoadjuvant chemotherapy, 16 received adjuvant chemotherapy, and 19 patients received salvage chemotherapy for recurrent disease. | 93 | Patients receiving neoadjuvant chemotherapy are excluded from the prognostic biomarker evaluation. Optionally, the user can exclude chemotherapy-treated patients from the prognostic biomarker evaluation. |
| Lindgren[[6](#_ENREF_6)]  (GSE19915) | 156 | Tumor profiles were obtained from 144 patients undergoing transurethral resection of the bladder from the University Hospital of Lund, Sweden, as well as normal profiles from 12 patients with non-bladder cancer related disorders. Forty-six patients are later cystectomized, the majority (N = 32) who had HG, MI tumors. | 156 | In the prognostic biomarker evaluation in patients with HG, MI tumors, 11 patients not cystectomized are excluded. Ninety patients are profiled in Lindgren-2 and are excluded if Lindgren-2 is analyzed. |
| Lindgren-2 [[7](#_ENREF_7)] (GSE32548) | 131 | Tumor profiles were obtained from 131 patients undergoing transurethral resection of the bladder from University Hospital of Lund, Sweden. Thirty-two patients are later cystectomized, the majority (N = 21) who had HG, MI tumors. | 131 | In the prognostic biomarker evaluation in patients with HG, MI tumors, 14 patients not cystectomized are excluded. |
| MDA-1 [[8](#_ENREF_8)]  (GSE48276) | 128 | Profiles were obtained from 128 bladder tumors from patients at MD Anderson (MDA) or Fox-Chase Cancer Center and Thomas Jefferson University (Philadelphia samples). Of the MDA tumor samples, only 24 had urothelial histology at cystectomy and were not treated by neoadjuvant chemotherapy | 22 | The Philadelphia samples were all MI and did not have survival information, and were therefore not analyzed. Of the MDA samples, tumors not having urothelial histology at cystectomy and those treated with neoadjuvant chemotherapy were excluded. Two additional NMI tumors were also excluded. |
| MDA-2 [[8](#_ENREF_8)]  (GSE48075) | 142 | Gene expression profiles from 142 primary bladder tumors were obtained. | 142 | No samples were excluded. |
| MSKCC [[9](#_ENREF_9)]  (S) | 157 | Gene expression profiles from 105 patients with bladder cancer were obtained along with normal samples, with some samples in duplicate or triplicate. | 91 | Previously processed data is included [[10](#_ENREF_10)], which averaged replicate profiles to produce a single gene expression profile for each patient and removed several low quality samples. |
| UVA[[11](#_ENREF_11)]  (GSE37317) | 19 | Gene expression profiles of 19 bladder cancer patients were obtained at the University of Virginia. | 18 | One sample with squamous cell histology was excluded. |
| Stransky-1 and Stransky-2[[12](#_ENREF_12)]  (E-TABM-147) | 103 | Gene expression profiles from 52 bladder cancer patients from Henri Mondor Hosptial are available, with many samples profiled in duplicate. Ten normal samples were also obtained. | 62 | Replicate profiles were averaged to produce a single gene expression profile for each patient |

**Table S2. Association of stage (MI vs. NMI) and grade (HG vs. LG) with the *best available* endpoint.** Statistically significant associations (P < 0.05) are highlighted in bold.

|  | Best available endpoint* | Stage | | | Grade | | |
| --- | --- | --- | --- | --- | --- | --- | --- |
|  |  | #NMI, MI | HR | Logrank P-value | #LG, HG | HR | Logrank P-value |
| Blaveri | OS | 25, 44 | 1.97 | 0.071 | 10, 57 | 2.28 | 0.164 |
| CNUH | DSS | **104, 61** | **17.90** | **< 0.001** | **105, 60** | **5.99** | **< 0.001** |
| DFCI-1 | OS | **15, 75** | **5.66** | **0.007** | 6, 84 | 3.57 | 0.178 |
| Lindgren | OS | **97, 43** | **12.83** | **< 0.001** | **72, 70** | **6.79** | **< 0.001** |
| Lindgren-2 | OS | **59, 29** | **13.24** | **< 0.001** | **40, 49** | **3.19** | **0.030** |
| MSKCC | DSS | **22, 65** | **14.6** | **< 0.001** | **17, 70** | **11.29** | **0.003** |

*The best available endpoint is defined as the first available endpoint in the following order: DSS, OS, RFS. Abbreviations: DSS, disease-specific survival; OS, overall survival; RFS, recurrence-free survival.

**References**

1. Dyrskjot L, Kruhoffer M, Thykjaer T, Marcussen N, Jensen JL, Moller K, Orntoft TF: **Gene expression in the urinary bladder: a common carcinoma in situ gene expression signature exists disregarding histopathological classification**. *Cancer research* 2004, **64**(11):4040-4048.

2. Dyrskjot L, Zieger K, Real FX, Malats N, Carrato A, Hurst C, Kotwal S, Knowles M, Malmstrom PU, de la Torre M *et al*: **Gene expression signatures predict outcome in non-muscle-invasive bladder carcinoma: a multicenter validation study**. *Clinical cancer research : an official journal of the American Association for Cancer Research* 2007, **13**(12):3545-3551.

3. Blaveri E, Simko JP, Korkola JE, Brewer JL, Baehner F, Mehta K, Devries S, Koppie T, Pejavar S, Carroll P *et al*: **Bladder cancer outcome and subtype classification by gene expression**. *Clinical cancer research : an official journal of the American Association for Cancer Research* 2005, **11**(11):4044-4055.

4. Kim WJ, Kim EJ, Kim SK, Kim YJ, Ha YS, Jeong P, Kim MJ, Yun SJ, Lee KM, Moon SK *et al*: **Predictive value of progression-related gene classifier in primary non-muscle invasive bladder cancer**. *Molecular cancer* 2010, **9**:3.

5. Riester M, Taylor JM, Feifer A, Koppie T, Rosenberg JE, Downey RJ, Bochner BH, Michor F: **Combination of a novel gene expression signature with a clinical nomogram improves the prediction of survival in high-risk bladder cancer**. *Clinical cancer research : an official journal of the American Association for Cancer Research* 2012, **18**(5):1323-1333.

6. Lindgren D, Frigyesi A, Gudjonsson S, Sjodahl G, Hallden C, Chebil G, Veerla S, Ryden T, Mansson W, Liedberg F *et al*: **Combined gene expression and genomic profiling define two intrinsic molecular subtypes of urothelial carcinoma and gene signatures for molecular grading and outcome**. *Cancer research* 2010, **70**(9):3463-3472.

7. Lindgren D, Sjodahl G, Lauss M, Staaf J, Chebil G, Lovgren K, Gudjonsson S, Liedberg F, Patschan O, Mansson W *et al*: **Integrated genomic and gene expression profiling identifies two major genomic circuits in urothelial carcinoma**. *PloS one* 2012, **7**(6):e38863.

8. Choi W, Porten S, Kim S, Willis D, Plimack ER, Hoffman-Censits J, Roth B, Cheng T, Tran M, Lee IL *et al*: **Identification of distinct basal and luminal subtypes of muscle-invasive bladder cancer with different sensitivities to frontline chemotherapy**. *Cancer cell* 2014, **25**(2):152-165.

9. Sanchez-Carbayo M, Socci ND, Lozano J, Saint F, Cordon-Cardo C: **Defining molecular profiles of poor outcome in patients with invasive bladder cancer using oligonucleotide microarrays**. *J Clin Oncol* 2006, **24**(5):778-789.

10. Smith SC, Baras AS, Dancik G, Ru Y, Ding KF, Moskaluk CA, Fradet Y, Lehmann J, Stockle M, Hartmann A *et al*: **A 20-gene model for molecular nodal staging of bladder cancer: development and prospective assessment**. *The Lancet Oncology* 2011, **12**(2):137-143.

11. Smith SC, Baras AS, Owens CR, Dancik G, Theodorescu D: **Transcriptional signatures of Ral GTPase are associated with aggressive clinicopathologic characteristics in human cancer**. *Cancer research* 2012, **72**(14):3480-3491.

12. Stransky N, Vallot C, Reyal F, Bernard-Pierrot I, de Medina SG, Segraves R, de Rycke Y, Elvin P, Cassidy A, Spraggon C *et al*: **Regional copy number-independent deregulation of transcription in cancer**. *Nature genetics* 2006, **38**(12):1386-1396.
