# Supplementary material for: An online tool for evaluating diagnostic and prognostic gene expression biomarkers in bladder cancer
Source: BMC Urol. 2015 Jul 1;15:59. doi: 10.1186/s12894-015-0056-z (PMC4487975; doi:10.1186/s12894-015-0056-z)
Supplement: Additional file 2: Figure S1. — Association of treatment with outcome in CNUH and DFCI cohorts. Kaplan-Meier curves for CNUH cohort showing association of disease-specific survival (DSS) and overall survival (OS) with (A) intravesical Bacillus Calmette-Guerin (BCG) therapy in patients with NMI tumors and (B) cisplatin-based adjuvant chemotherapy in patients with MI tumors. C, Association of recurrence-free survival (RFS) with adjuvant chemotherapy in patients with MI tumors in DFCI chort. P-values are calculated by log-rank test. Abbreviations: HR, hazard ratio; N+, nodal involvement (pN1-pN3); M+, distant metastasis. [file 12894_2015_56_MOESM2_ESM.pptx]

## Slide 1
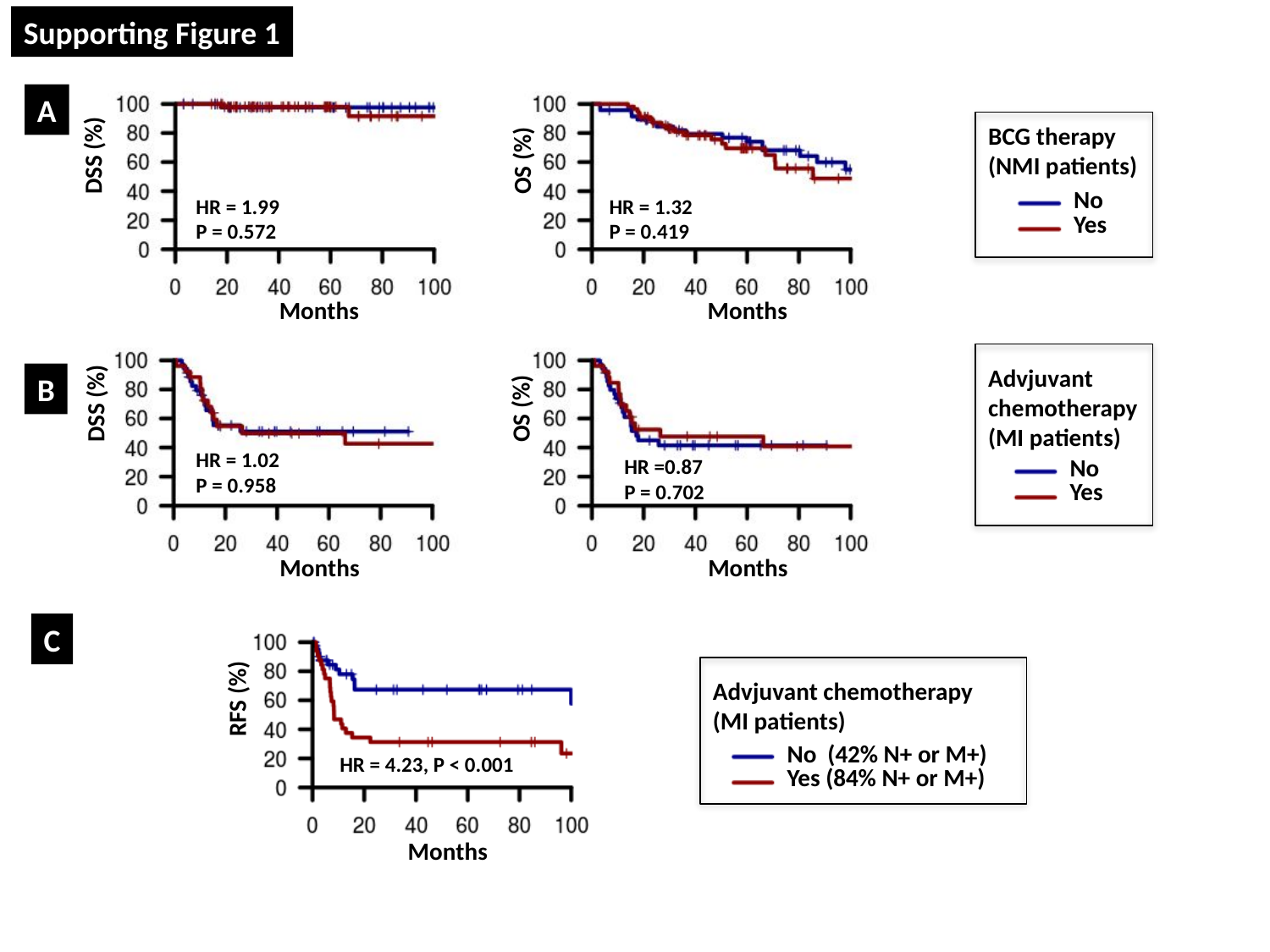

Supporting Figure 1
A
BCG therapy
(NMI patients)
DSS (%)
OS (%)
No
HR = 1.99
P = 0.572
HR = 1.32
P = 0.419
Yes
Months
Months
Advjuvant chemotherapy
(MI patients)
B
DSS (%)
OS (%)
HR = 1.02
P = 0.958
No
HR =0.87
P = 0.702
Yes
Months
Months
C
Advjuvant chemotherapy
(MI patients)
RFS (%)
No (42% N+ or M+)
HR = 4.23, P < 0.001
Yes (84% N+ or M+)
Months
